# Direct H-He chemical association in superionic FeO_2_H_2_He at deep-Earth conditions

**DOI:** 10.1093/nsr/nwab168

**Published:** 2021-09-02

**Authors:** Jurong Zhang, Hanyu Liu, Yanming Ma, Changfeng Chen

**Affiliations:** International Center for Computational Method and Software & State Key Laboratory of Superhard Materials & Key Laboratory of Physics and Technology for Advanced Batteries (Ministry of Education), College of Physics, Jilin University, Changchun 130012, China; Shandong Provincial Engineering and Technical Center of Light Manipulations & Shandong Provincial Key Laboratory of Optics and Photonic Device, School of Physics and Electronics, Shandong Normal University, Jinan 250358, China; International Center for Computational Method and Software & State Key Laboratory of Superhard Materials & Key Laboratory of Physics and Technology for Advanced Batteries (Ministry of Education), College of Physics, Jilin University, Changchun 130012, China; International Center of Future Science, Jilin University, Changchun 130012, China; International Center for Computational Method and Software & State Key Laboratory of Superhard Materials & Key Laboratory of Physics and Technology for Advanced Batteries (Ministry of Education), College of Physics, Jilin University, Changchun 130012, China; International Center of Future Science, Jilin University, Changchun 130012, China; Department of Physics and Astronomy, University of Nevada, Las Vegas, NV 89154, USA

**Keywords:** high pressure, crystal structures, molecular dynamics, hydrogen-helium chemistry

## Abstract

Hydrogen and helium are known to play crucial roles in geological and astrophysical environments; however, they are inert toward each other across wide pressure-temperature (P-T) conditions. Given their prominent presence and influence on the formation and evolution of celestial bodies, it is of fundamental interest to explore the nature of interactions between hydrogen and helium. Using an advanced crystal structure search method, we have identified a quaternary compound FeO_2_H_2_He stabilized in a wide range of P-T conditions. *Ab initio* molecular dynamics simulations further reveal a novel superionic state of FeO_2_H_2_He hosting liquid-like diffusive hydrogen in the FeO_2_He sublattice, creating a conducive environment for H-He chemical association, at P-T conditions corresponding to the Earth's lowest mantle regions. To our surprise, this chemically facilitated coalescence of otherwise immiscible molecular species highlights a promising avenue for exploring this long-sought but hitherto unattainable state of matter. This finding raises strong prospects for exotic H-He mixtures inside Earth and possibly also in other astronomical bodies.

## Teaser

Prediction evidenced a chemical association of hydrogen and helium facilitated by their reaction with iron peroxide in forming a rare quaternary compound FeO_2_H_2_He, raising strong prospects for exotic H-He mixtures inside Earth and possibly also in other astronomical bodies.

## INTRODUCTION

The recent discovery of iron peroxide FeO_2_ and its reaction with prominent elements and compounds at pressure-temperature (P-T) conditions corresponding to Earth's core-mantle boundary (CMB) [1–5] has opened exciting avenues for exploring new materials [6,7] and properties that have great impact on major geophysical and geochemical phenomena. Of particular significance are FeO_2_-derived minerals that help elucidate distinct pathways for hydrogen and oxygen cycles as well as mechanisms for water transportation deep inside the Earth's interior, which are crucial to understanding key geological events, especially those vital to creating the conditions for life on Earth [8]. Further studies [9,10] revealed mechanisms for structural formation and evolution of a dense phase of FeO_2_H at CMB P-T conditions. Moreover, a recent work [7] predicted a rare helium-bearing compound FeO_2_He that is robust and viable in Earth's CMB region, offering a compelling scenario for elucidating the origin of enigmatic deep-Earth reservoirs for primordial helium inferred by geochemical evidence [11–14]. The unique ability of FeO_2_ to bond with hydrogen and helium and form new compounds has major implications for understanding the evolution and dynamics of Earth and other giant solar and extrasolar planets.

Hydrogen and helium are the most abundant elements in the universe and play crucial roles in geological and astrophysical environments, but they are known to be inert toward each other across wide P-T and concentration ranges and remain largely immiscible up to multi-megabar pressures and 3000–4000 K temperatures [15–22]. Given their prominent presence and influence on the formation and evolution of celestial bodies, it is of great interest and significance to explore and decipher the nature of interactions between hydrogen and helium, especially possible chemical association that would have considerable impacts in many scientific fields, from chemistry, physics, geoscience to astrophysics [23–25]. Recent studies have identified compounds that host both hydrogen and helium, such as in highly compressed helium-water compounds [26–29], but there is no evidence of direct chemical association of these two elements. A recent experimental study [30] probed phase diagram of dense H_2_-He mixtures and reported evidence of strong chemical association of the constituent molecules at pressures < 75 GPa; but later experiments presented opposing conclusions [31,32], showing no evidence for chemical association in H_2_-He mixtures up to 250 GPa at 300 K and pointing to N_2_ contamination as the source for the previously observed phenomena [30]. The quest to seek possible reactivity of hydrogen and helium remains an intriguing open challenge.

In this work, we present evidence from first-principles calculations showing direct and prevalent chemical association of hydrogen and helium facilitated by their reaction with iron peroxide FeO_2_ in forming a rare quaternary compound FeO_2_H_2_He. We conducted extensive crystal structure searches and evaluated the Gibbs free energy of the three reactants and the resulting product to determine their relative phase stability; we also performed *ab initio* molecular dynamics (AIMD) simulations to assess temperature-driven partial melting of the compound. The results show that FeO_2_H_2_He is viable in a large region of the P-T phase diagram. Most interestingly, in a wide swath of the phase space corresponding to Earth's lowest-mantle regions, this quaternary compound stays in a superionic state hosting liquid-like hydrogen inside the FeO_2_He sublattice that remains intact in crystal form. This exotic solid-liquid mixture state of matter makes an unusually conducive environment, promoting close coalescence of hydrogen and helium. An analysis of the H-He radial distribution function (RDF) and bonding features in superionic FeO_2_H_2_He reveals significant direct H-He chemical association as indicated by RDF peaks at short distances with considerable bonding as revealed by interatomic charge characters. These results highlight a compelling case of H-He chemical association, which may be harbored in deep-Earth regions, and the constructed phase diagram provides crucial guidance for exploring solid and superionic phases of FeO_2_H_2_He in laboratory experiments and for modeling interiors of giant solar and extrasolar planets. The computational approach adopted in this work in search of stable quaternary compounds represents the state of the art in the very active and diverse research area of crystal structure prediction, and our reported results may help resolve a significant scientific problem of long-standing and broad interest in many fields of chemistry, physics, geophysical and planetary sciences among others.

## RESULTS AND DISCUSSIONS

**Figure 1.**
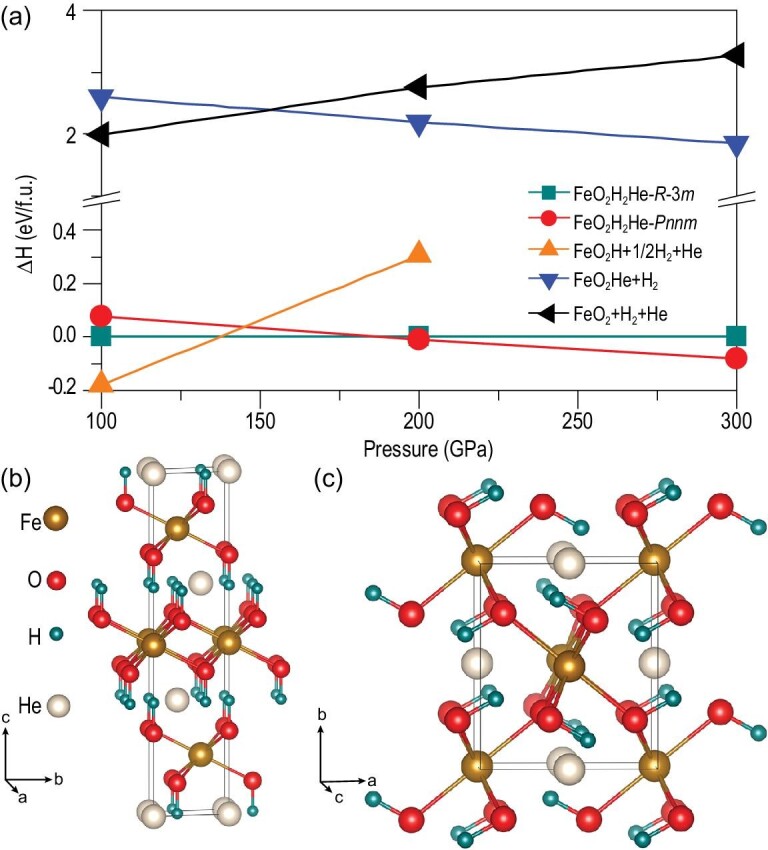
Enthalpy and structures of FeO_2_H_2_He. (a) Calculated enthalpy of the two FeO_2_H_2_He crystal structures compared with several probable decomposition products (FeO_2_, FeOOH, FeOOHe, H and He) in the pressure range of 100–300 GPa. The predicted FeO_2_H_2_He structure in the (b) *R*-3*m* symmetry and (c) *Pnnm* symmetry.

Our first-principles crystal structure search identified a quaternary compound with stable stoichiometry, FeO_2_H_2_He. Calculated enthalpy data [Fig. 1(a)] show that this new compound adopts a crystal structure in the *R*-3*m* symmetry [Fig. 1(b)] at lower pressures and stabilizes relative to the reactants FeO_2_H + H + He above 136 GPa; at further increased pressures over 188 GPa, this compound transitions into a structure in the *Pnnm* symmetry [Fig. 1(c)]. Details on the crystal (see Supplementary Table 1) and electronic structures (see Supplementary Fig. 1) are provided in the Supplemental data. While these predicted structures have not been checked exhaustively against all possible decompositions because of computational constraints, they have been extensively examined to assess their viability. A full search process has established that this compound is the most stable in the Fe-O-H-He quaternary compositional space and, crucially, energetically favorable compared to the expected reactants, as indicated in Fig. 1(a). Moreover, we performed phonon calculations, and the results showed that there are no imaginary modes in the entire Brillouin zone from 100 GPa to 300 GPa (see Supplementary Fig. 2), thus confirming the dynamical stability of the predicted FeO_2_H_2_He structures. Results in Fig. 1 show that the sublattice of the Fe-O-H framework in FeO_2_H_2_He is identical to that of the *P-*3*m*1 phase of FeO_2_H_2_. The FeO_6_H_6_ units in FeO_2_H_2_He also exist in M(OH)_2_ (M = Ca, Mg, Ni, Co, Mn *et al.*) compounds [33–36], which indicates the broad feasibility of inserting He atoms into these M(OH)_2_ compounds. It is noted that the inclusion of the van der Waals interactions causes some quantitative changes in the calculated transition pressures (see Supplementary Fig. 3), but does not change the main conclusion of this work.

**Figure 2.**
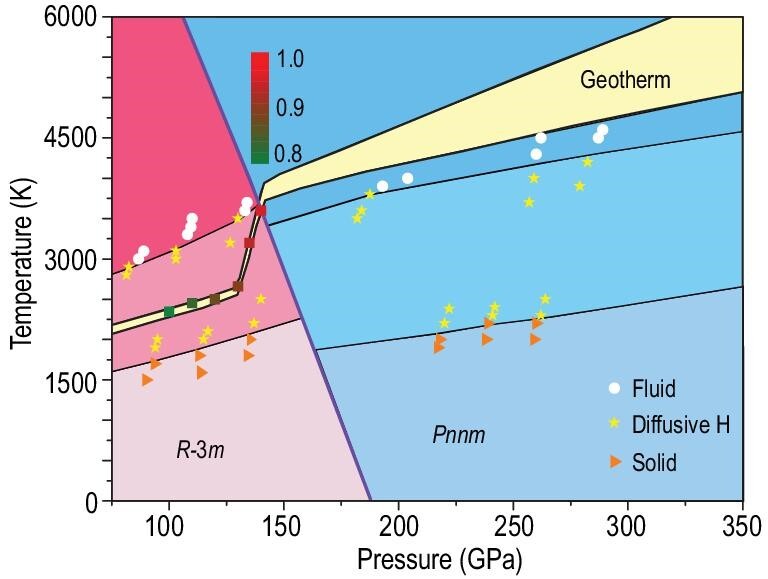
Pressure-temperature phase diagram of FeO_2_H_2_He determined by Gibbs free energy from first-principles calculations and mean-square atomic displacements from AIMD simulations. The phase boundary is marked by the thick black line and different structures within each phase are determined based on the AIMD results, where triangles, stars and circles represent, respectively, the solid, superionic (with diffusive H atoms) and liquid structures. Also presented are the phase coexistence probabilities, as indicated by the colored scale bar shown in the inset, in the context of the Boltzmann distribution for the FeO_2_H_2_He structures relative to the dehydrogenation products FeO_2_H, H_2_ and He at selected points along the geotherm in the pressure range of 100–140 GPa. The yellow region presents the geotherm of the Earth's core from Ref. [39].

**Figure 3.**
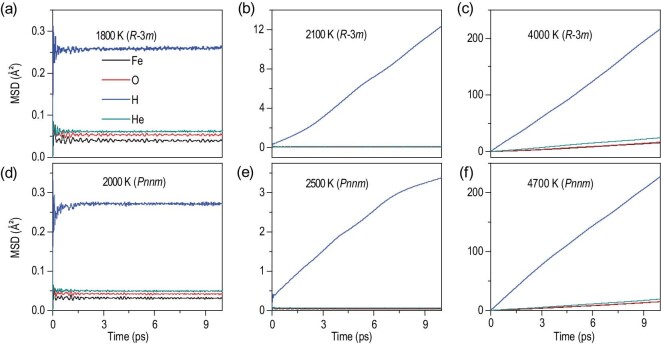
Mean-square displacements (MSDs) of atoms from AIMD simulations. (a–c) For the *R*-3*m* phase, there is no diffusion for any atom at 1800 K and 134 GPa in the solid structure; H atoms are diffusive at 2100 K and 136 GPa in the superionic structure; all atoms become diffusive at 4000 K and 170 GPa in the liquid structure. (d–f) For the *Pnnm* phase, there is no diffusion for any atom at 2000 K and 258 GPa in the solid structure; H atoms are diffusive at 2500 K and 263 GPa in the superionic structure; all atoms become diffusive at 4700 K and 306 GPa in the liquid structure.

In assessing fundamental behaviors of the newly identified FeO_2_H_2_He crystal phases, a crucial task is to establish their P-T phase diagram. To this end, we performed two sets of calculations. First, we calculated phonon dispersions and the corresponding phonon density of states (PDOS) within the harmonic approximation as implemented in the PHONOPY code [37], and then used the obtained PDOS as input to evaluate the vibrational contribution to the entropy of each phase. Combining with the total internal energy, pressure and volume determined by first-principle calculations, we obtained for both the *R*-3*m* and *Pnnm* phases their Gibbs free energy, which allow determination of the phase boundary as shown in Fig. 2. Second, we performed AIMD simulations for the two FeO_2_H_2_He phases in the pressure range of 70–300 GPa over a temperature range of 0–6000 K to evaluate thermodynamic stability and temperature driven diffusive and melting behaviors by examining mean square displacements (MSDs) of the Fe, O, H and He atoms in the crystal structures. Some representative MSD data are presented in Fig. 3 to showcase the typical behaviors and illustrate the assessment process and criteria. Following this procedure that has been applied to sufficient P-T points, we obtained the boundaries, as indicated in the phase diagram shown in Fig. 2, between the three distinct states: (i) solid, where all atoms remain near their equilibrium crystal lattice sites, (ii) diffusive H, where H atoms show significant deviations from their equilibrium sites while all other atoms remain near their equilibrium sites, and (iii) fluid, where all atoms deviate from their equilibrium sites. In all the simulated cases, increasing temperature first drives H atoms off their equilibrium sites into a diffusive phase, followed by diffusive behaviors of all other atoms that happen almost concurrently, signifying the melting of the crystal structure into the fluid phase. It is noted that the predicted FeO_2_H_2_He structures may coexist with the dehydrogenation products FeO_2_H, H_2_ and He toward the low-pressure side of the P-T phase diagram shown here; however, results from AIMD simulations show that the FeO_2_H_2_He phases are at least metastable and there is no immediate phase transition to another FeO_2_H_2_He structure in the P-T space studied here as indicated by the stable atomic positions (see below). We adopted a Boltzmann distribution description for phase coexistence probability [38] of FeO_2_H_2_He relative to its dehydrogenation products at selected pressures along the geotherm [39] (see Fig. 2), and the results indicate robust presence of FeO_2_H_2_He in the P-T regions of interest.

We show that each of the solid, diffusive H, and fluid states of the two FeO_2_H_2_He phases has a large stability field in the P-T phase diagram (Fig. 2), determined by extensive energetic (Gibbs free energy), dynamic (phonon dispersion) and thermodynamic (AIMD) calculations. It is noted that the stability field of the diffusive H state of the *R*-3*m* phase includes the Earth's geotherm in the vast lower-mantle region, covering the core-mantle boundary (CMB), indicating that the superionic state of this extraordinary quaternary compound may exist in Earth's lower-mantle regions. Moreover, this superionic state, in which H atoms move in a liquid-like state in the still intact solid Fe-O-He sublattice, greatly enhances the probability of bonding interactions of H atoms with other atoms in the compound, especially the long-sought H-He chemical association. To explore this intriguing prospect, we performed extensive computational studies and systematic analysis of the H-He bonding in FeO_2_H_2_He under pertinent P-T conditions. We calculated RDFs from AIMD simulation data to assess interatomic distances in the solid and superionic phases, along with an examination of select short bond lengths. We also performed a systematic evaluation of the electronic density and the Laplacian of the electronic density at the bond critical point, which is the saddle point along neighboring bond paths for interatomic charge distribution commonly used in characterization of bonding in the context of QTAIM [40].

**Figure 4.**
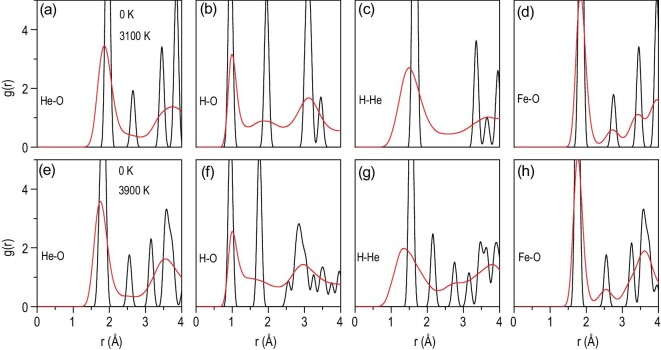
Radial distribution functions (RDFs) g(r). G(r) at 0 K, 120 GPa and 3100 K, 147 GPa for the *R-*3*m* phase (a–d), and at 0 K, 240 GPa and 3900 K, 279 GPa for the *Pnnm* phase (e–h).

We first set a key reference by examining the bonding and charge states of the two solid FeO_2_H_2_He phases at representative pressure points at 0 K. The H-He bond length in the *R*-3*m* phase at 100 GPa is 1.67 Å and the value for the *Pnnm* phase at 300 GPa is 1.50 Å. While these bonds are considerably shorter than the sum of the van der Waals radii for H and He atom (2.50 Å), a QTAIM analysis (see results in Table 1 and discussions below) showed no evidence of H-He bonding. Calculated RDFs (Fig. 4) exhibited sharp peaks reflecting well defined and separated interatomic distances in these solids.

**Table 1.** H-He bond length d*_H-He_* (in Å), electron density *ρ* (in e^–^/Å^3^) and its Laplacian (in e^–^/Å^–5^) at bond critical points of FeO_2_H_2_He at selected P-T points. Results for conventional hydrogen bonding [41] are listed as reference.

BCP name	d_H-He_(Å)	*Ρ*	Δ^2^*ρ*
Hydrogen bond		0.04–0.24	0.58–3.35
100 GPa, 0 K (*R*-3*m*)	1.67	None	None
300 GPa, 0 K (*Pnnm*)	1.50	None	None
147 GPa, 3100 K (*R*-3*m*)	0.92	1.11	0.72
	1.04	0.85	10.68
	1.08	0.81	10.10
	1.21	0.51	5.92
	1.24	0.52	6.40
	1.25	0.47	5.46
	1.26	0.45	5.02
	1.27	0.45	4.65
	1.29	0.41	4.70
	1.29	0.43	4.21
	1.29	0.51	10.2
	1.30	0.43	7.30
279 GPa, 3900 K (*Pnnm*)	0.97	1.14	15.08
	1.07	0.78	8.05
	1.13	0.67	7.86
	1.13	0.75	11.36
	1.14	0.74	6.68
	1.17	0.63	7.48
	1.20	0.67	11.72
	1.24	0.51	7.24
	1.25	0.50	8.04
	1.26	0.56	10.62
	1.30	0.49	10.13

At elevated temperatures and pressures pertinent to deep-Earth regions, the RDFs show considerable thermal fluctuations (Fig. 4), indicating greatly enhanced ranges of atomic movements. While all the RDFs are thermally broadened, those involving H exhibit more pronounced changes because of the diffusive nature of the H atoms in the superionic phases of FeO_2_H_2_He. In particular, the H-He RDFs undergo the most dramatic broadening, with substantial shifts of the H-He RDF weights to the lower distances. Such remarkably reduced interatomic separations suggest higher probability for H-He bonding interactions. To verify this scenario, we examined the AIMD simulation data and found significant bonding features in the two superionic phases when the H-He bond length is shorter than 1.3 Å, as indicated by the electronic charge density *ρ* and the Laplacian of the charge density Δ^2^*ρ* at the bond critical point where key bonding characters are evaluated in QTAIM to probe the formation of interatomic bonding [40]. In Table 1, we present pertinent results that showcase appreciable H-He bonding comparable to conventional hydrogen bonding [41], which offers compelling quantitative evidence for direct H-He chemical association in superionic FeO_2_H_2_He. Our analysis reveals that the H-He pairs with clear bonding features comprise a large fraction (up to 20–40%) of the total number of H-He pairs in the AIMD simulation cells that fall under the first RDF peaks for the respective superionic phases. This phenomenon indicates a strong tendency for H-He bonding formation in FeO_2_H_2_He at P-T conditions pertinent to deep-Earth environments.

It is noted that the charge analysis performed on a snapshot taken from the MD simulation can be considered representative of the system in a time averaged sense. The comparison with the stable hydrogen bonding is meant to provide a relative reference and measure with an established bonding configuration, with the understanding that the charge configurations in FeO_2_H_2_He are dynamic and any given specific ‘bonding’ is only transient in nature. On this basis, the present analysis of the dynamic bonding provides a useful description of the superionic state of FeO_2_H_2_He, especially the degree of H-He interaction in such a novel state over a wide swath of the P-T space. We performed calculations for insights into the dynamic charge distribution in FeO_2_H_2_He. The calculated Crystal Orbital Hamilton Populations (COHPs) (see Supplementary Fig. 4) show that FeO_2_H_2_He at 147 GPa and 3100 K host anti-bonding states, and there are overlaps between the H 1*s* and He 1*s* states, indicating charge transfer from the H to He atoms. Moreover, we analyzed the data from the molecular dynamics simulations on the nearest H-He distance of 0.9–1.3 Å, and the results (see Supplementary Fig. 5) indicate that the lifetime for such bonding states is around 0.01 ps.

## CONCLUSION

In summary, we identified a novel quaternary compound FeO_2_H_2_He using an advanced crystal structure search approach and established its P-T phase diagram from extensive first-principles calculations of structural, phono, and molecular dynamics. This compound adopts three distinct phases: solid, superionic with diffusive H and liquid. Each of them occupies a large region in the phase diagram. The superionic phase is viable under the conditions in Earth's vast lower mantle, which includes the enigmatic CMB region where pertinent reactants have been predicted to be present. The superionic nature of this new compound promotes unprecedented close coalescence of hydrogen and helium in a chemically facilitated environment. These findings provide crucial knowledge for deciphering the long-sought H-He chemical association in a large swath of P-T phase space previously thought to be off-limits for H-He miscibility, offering new insights for elucidating novel physics and chemistry of the predicted compound FeO_2_H_2_He in a wide range of high P-T conditions and also for modeling solar and extrasolar giant gas planets.

## METHODS

### Structural predictions

Crystal-structure searches for quaternary compounds are computationally demanding; our task here is facilitated by knowing the constituent reactants, namely iron peroxide, hydrogen and helium, which have been predicted to exist in Earth's lowest-mantle regions through geophysical and geochemical processes [1–5,7,11–14]. Accordingly, we have explored Fe-O-H-He compositional space with energetic evaluations in relation to FeO_2_, H_2_ and He to determine relative structural phase stability.

Our structure search is based on global optimization of potential-energy surfaces using the CALYPSO methodology [42,43], which has been successfully employed in predicting a large variety of crystal structures [6,44,45]. Evolutionary variable-cell calculations were performed at 100, 200 and 300 GPa with 1, 2, 3, and 4 FeO_2_H_2_He formula units per cell, retaining 60% lowest-enthalpy structures to produce the next-generation structures by a particle swarm optimization procedure and generating the remaining 40% structures randomly within the symmetry constraint. The number of atoms in the unit cell is in the range of 20–30 for most of the reported structure searching simulations. In light of several Fe-O compounds predicted at 100 GPa and 300 GPa [1,46], we have particularly performed extensive structure searches on many relevant and viable compositions, e.g. FeO-H-He, FeO_2_-H-He, Fe_2_O_3_-H-He, Fe_3_O_4_-H-He and FeO_3_-H-He at 100 GPa and Fe_3_O-H-He, Fe_2_O-H-He, FeO-H-He and FeO_2_-H-He at 300 GPa (see Supplementary Table 2 and Supplementary Table 3 for details). Most searches converge in 30–40 generations with about 1000 structures generated; in all, we have examined about 227 000 structures in this study.

### *Ab initio* calculations

First-principles total-energy and electronic property calculations were carried out using density functional theory (DFT) as implemented in the VASP code [47], adopting the frozen-core all-electron projector-augmented wave (PAW) method [48], with 3*s*^2^3*p*^6^3*d*^7^4*s*^1^, 2*s*^2^2*p*^4^, 1*s*^1^ and 1*s*^2^ treated as valence electrons for Fe, O, H and He, respectively, and the Perdew-Burke-Ernzerhof (PBE) exchange-correlation functional in the generalized gradient approximation (GGA) [49,50]. Correlation effects among the Fe 3*d* electrons were treated in the GGA + U approach [51,52], adopting the recently proposed on-site Coulomb interaction U = 5.0 eV and a Hund's coupling J = 0.8 eV [53]. A cutoff energy of 1000 eV for the plane-wave expansion and fine Monkhorst-Pack k-meshes [54] were chosen to ensure enthalpy convergence of several meV/atom.

### ZPE, phase-diagram and phonon calculations

To properly describe the quantum effect in hydrogen and helium, we included the zero-point energy (ZPE) in all energetic calculations. To determine the phase boundaries between the solid phases, we performed calculations to determine the Gibbs free energy *G* = *U* + *PV − TS*, where *U*, *P*, *V*, *T* and *S* are the internal energy, pressure, volume, temperature and entropy, respectively. The vibrational energy and entropy were obtained from lattice dynamic calculations using the quasi-harmonic approximation as implemented in the PHONOPY code. Phonon calculations were carried out using a supercell approach [55] as implemented in PHONOPY code [37].

### Bonding and molecular dynamics calculations

We performed bonding analysis using the quantum theory of atoms in molecules (QTAIM) approach [40]. We also performed *ab initio* molecular dynamics (AIMD) simulations in the *NVT* (*N*-particle, *V*-volume, *T*-temperature) ensemble implemented in the VASP code to probe temperature-driven diffusive and melting behaviors. We employed a simulation time of 10 ps with a time step of 0.5 fs and a 3 times supercell containing 162 atoms in the *R*-3*m* phase and a 2 times supercell containing 144 atoms in the *Pnnm* phase.

## Supplementary Material

nwab168_Supplemental_File
