# Supplementary material for: Direct H-He chemical association in superionic FeO2H2He at deep-Earth conditions
Source: Natl Sci Rev. 2021 Sep 2;9(7):nwab168. doi: 10.1093/nsr/nwab168 (PMC9344844; doi:10.1093/nsr/nwab168)
Supplement: nwab168_Supplemental_File [file nwab168_supplemental_file.docx]

**Supplementary Data**

**Direct H-He Chemical Association in Superionic FeO_2_H_2_He**

**at Deep-Earth Conditions**

Zhang *et al.*

**Supplementary Table 1.** Calculated structural data of *R-*3*m* and *Pnnm* FeO_2_H_2_He phases using GGA+U functional.

| Phase | Pressure | Lattice Parameters | Atomic Coordinates | |
| --- | --- | --- | --- | --- |
| Atom x y z | | | | |
| *R-*3*m* | 100 GPa | a=2.893 Å, b=2.893 Å  c=11.037 Å  *α*=90°, *β*=90°, *γ*=90° | | Fe (3b) 0.000 0.000 0.500  O (6c) 0.000 0.000 0.243  H (6c) 0.000 0.000 0.332  He (3a) 0.000 0.000 0.000 |
| *Pnnm* | 300 GPa | a=3.650 Å, b=4.261 Å  c=2.625 Å  *α*=90°, *β*=90°, *γ*=120° | | Fe (2b) 0.000 0.000 0.500  O (4g) 0.126 0.754 0.000  H (4g) 0.893 0.646 0.000  He (2d) 0.000 0.500 0.500 |

**Supplementary Table 2.** The compositions and formular units of the Fe-O-H-He systems examined at 100 GPa.

| FeOHHe  (1, 2, 4 f.u.) | FeO_2_HHe  （1, 2, 4 f.u.） | FeO_2_H_4_He_3_  (1, 2 f.u.) | (FeO_2_)_2_H_4_He  (1, 2 f.u.) | FeO_3_H_4_He_2_  (1 f.u.) | Fe_2_O_3_H_2_He_2_  (1, 2 f.u.) |
| --- | --- | --- | --- | --- | --- |
| FeOH_2_He  (1, 2, 4 f.u.) | FeO_2_H_2_He  (1, 2, 3, 4 f.u.) | FeO_2_HHe_4_  (1, 2 f.u.) | (FeO_2_)_2_H_4_He_3_  (1 f.u.) | FeO_3_H_5_He  (1, 2 f.u.) | Fe_2_O_3_H_3_He_2_  (1, 2 f.u.) |
| FeOH_3_He  (1, 2, 4 f.u.) | FeO_2_HHe_2_  （1, 2, 4 f.u.） | FeO_2_H_2_He_4_  (1, 2 f.u.) | (FeO_2_)_3_H_2_He_2_  (1 f.u.) | FeO_3_H_6_He  (1, 2 f.u.) | Fe_2_O_3_H_2_He  (1, 2 f.u.) |
| FeOH_3_He_2_  (1, 2, 4 f.u.) | FeO_2_H_2_He_2_  （1, 2, 4 f.u.） | FeO_2_H_4_He_2_  (1, 2 f.u.) | (FeO_2_)_3_H_3_He  (1, 2 f.u.) | FeO_3_HHe  (1, 2 f.u.) | Fe_2_O_3_H_3_He  (1, 2 f.u.) |
| FeOHHe_2_  (1, 2, 4 f.u. ) | FeO_2_H_3_He  （1, 2 f.u.） | FeO_2_H_4_He_3_  (1, 2 f.u.) | (FeO_2_)_3_HHe_2_  (1, 2 f.u.) | Fe_3_O_4_HHe  (1, 2 f.u.) | Fe_2_O_3_HHe  (1, 2, 4 f.u.) |
| FeOHHe_3_  (1, 2, 4 f.u.) | FeO_2_H_3_He_2_  （1, 2 f.u.） | FeO_2_H_6_He  (1, 2 f.u.) | FeO_3_H_2_He  (1 f.u.) | Fe_3_O_4_H_2_He  (1, 2 f.u.) | Fe_2_O_3_HHe_2_  (1, 2, 4 f.u.) |
| FeOH_5_He  (1, 2 f.u.) | FeO_2_HHe_3_  (1, 2, 4 f.u.) | FeO_2_H_7_He  (1 f.u.) | FeO_3_H_3_He  (1, 2 f.u.) | Fe_2_O_3_H_2_He_2_  (1, 2 f.u.) | Fe_2_O_3_H_5_He  (1 f.u.) |
| FeOH_4_He  (1 f.u.) | FeO_2_H_4_He  (1, 2, 4 f.u.) | FeO_2_H_8_He  (1 f.u.) | FeO_3_H_3_He_2_  (1 f.u.) | Fe_2_O_3_H_3_He_3_  (1 f.u.) | Fe_2_O_3_H_5_He_2_  (1 f.u.) |
| FeOH_5_He_2_  (1 f.u.) | (FeO_2_)_2_HHe  (1, 2, 4 f.u.) | (FeO_2_)_2_HHe_2_  (1, 2 f.u.) | FeO_3_H_3_He_3_  (1, 2 f.u.) | Fe_2_O_3_H_4_He  (1, 2 f.u.) | Fe_2_O_3_H_5_He_3_  (1 f.u.) |
| FeOH_5_He_3_  (1 f.u.) | FeO_2_H_2_He_3_  (1, 2, 4 f.u.) | (FeO_2_)_2_H_3_He_2_  (1, 2 f.u.) | FeO_3_H_3_He_4_  (1, 2 f.u.) | Fe_2_O_3_H_5_He  (1 f.u.) | Fe_2_O_3_H_6_He  (1 f.u.) |

**Supplementary Table 3.** The compositions and formula units of the Fe-O-H-He systems examined at 300 GPa.

| Fe_3_OHHe  (2 f.u.) | Fe_3_OH_2_He  (2 f.u.) | Fe_3_OH_3_He  (2 f.u.) | Fe_3_OH_4_He  (2 f.u.) | Fe_3_OHHe_2_  (2 f.u.) | Fe_3_OHHe_3_  (2 f.u.) |
| --- | --- | --- | --- | --- | --- |
| Fe_2_OHHe  (2 f.u.) | Fe_2_OH_2_He  (2 f.u.) | Fe_2_OH_2_He_2_  (2 f.u.) | Fe_2_OH_3_He  (2 f.u.) | Fe_2_OH_4_He  (2 f.u.) | Fe_2_OHHe_2_  (2 f.u.) |
| Fe_2_OHHe_3_  (2 f.u.) | FeOHHe  (2 f.u.) | FeOHHe_2_  (2 f.u.) | FeOH_2_He_2_  (2 f.u.) | FeOH_3_He  (2 f.u.) | FeOH_4_He  (2 f.u.) |
| FeOHHe_2_  (2 f.u.) | FeOHHe_3_  (2 f.u.) | FeO_2_HHe  (2 f.u.) | FeO_2_H_2_He  (2 f.u.) | FeO_2_H_2_He_2_  (2 f.u.) | FeO_2_H_3_He  (2 f.u.) |
| FeO_2_H_4_He  (2 f.u.) | FeO_2_HHe_2_  (2 f.u.) | FeO_2_HHe_3_  (2 f.u.) |  |  |  |





**Supplementary Figure 1| Calculated electronic band structures of FeO_2_H_2_ He.** Band structures for (a) the *R-*3*m* phase at 100 GPa and (b) the *Pnnm* phase at 300 GPa.





**Supplementary Figure 2| Calculated phonon dispersion curves of FeO_2_H_2_He.** Phonon dispersion at 100, 200, and 300 GPa for (a-c) the *R-*3*m* phase and (d-f) the *Pnnm* phase.


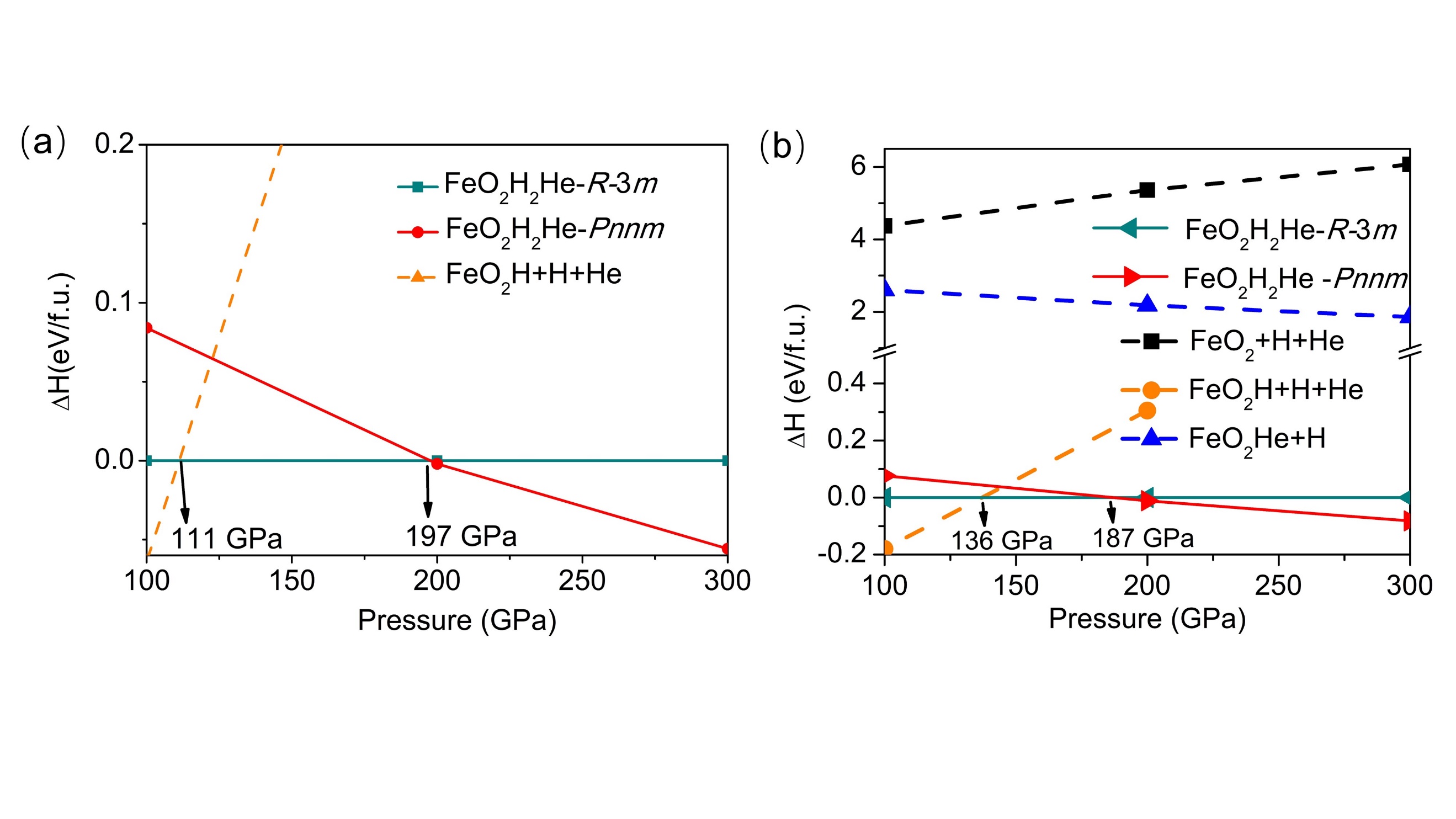


**Supplementary Figure 3|** **Enthalpy and structures of FeO_2_H_2_He.** Calculated enthalpy of the two FeO_2_H_2_He crystal structures compared with FeOOH+H+He in the pressure range of 100-300 GPa. (a) with the vdW (opt88-vdW) corrections and (b) without the vdW corrections.


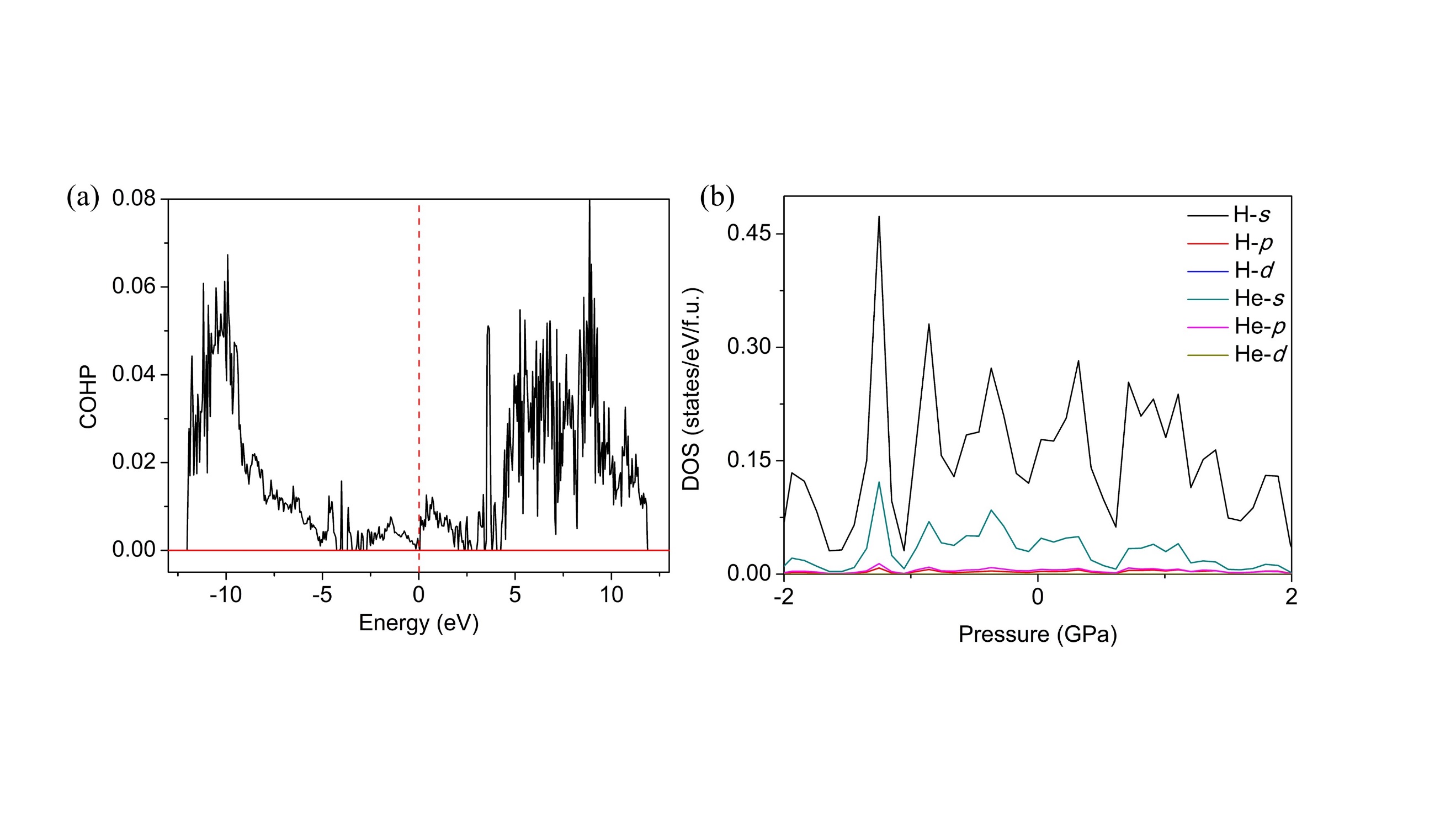


**Supplementary Figure 4|** (a) The COHP of H-He of FeO_2_H_2_He at 147 GPa and 3,100 K. (b) The projected electronic state density of H-He of FeO_2_H_2_He at 147 GPa.


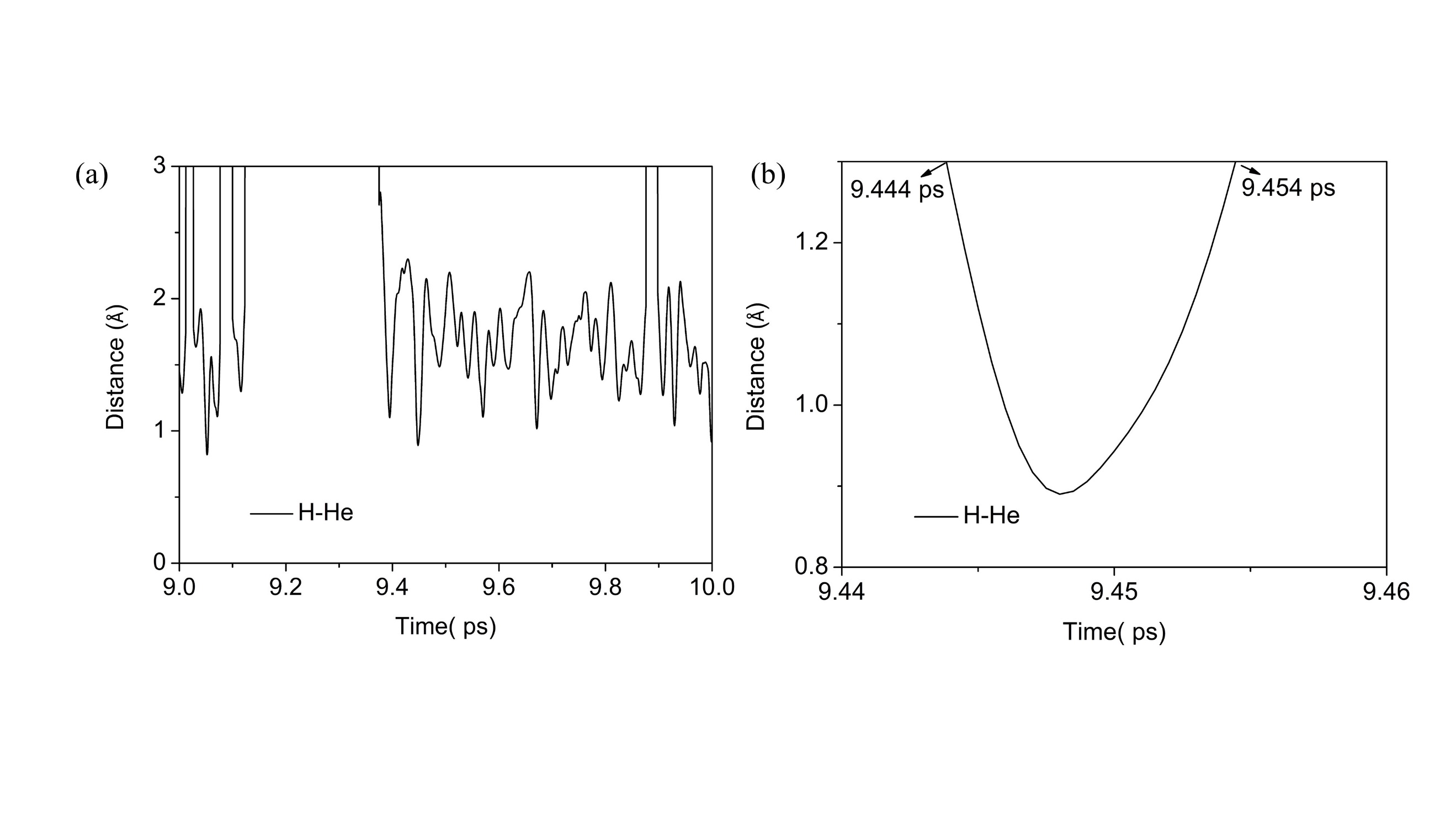


**Supplementary Figure 5|** The distance of H-He of FeO_2_H_2_He at 147 GPa and 3,100 K. (a) from 0.9 ps to 10 ps. (b) from 9.444 ps to 9.454 ps.
